# The uptake and utility of a reflective practice group for psychiatry registrars in a state-wide forensic service

**DOI:** 10.1177/10398562241277457

**Published:** 2024-09-13

**Authors:** Graham Walker, Jaydip Sarkar, Ruchi Bhalla

**Affiliations:** NHS Greater Glasgow and Clyde, Glasgow, UK; and University of Glasgow, Glasgow, UK; Sarkar Mental Health Experts Pty Ltd, Melbourne, VIC, Australia; 170472 RANZCP, Adelaide, SA, Australia

**Keywords:** Balint, reflective practice, qualitative, registrar, forensic

## Abstract

**Objectives:**

It is essential that mental health clinicians have access to reflective processes where they may understand and make sense of emotional responses to patients, teams, and organisations. The authors share their experience of initiating and successfully running a reflective practice group, framed with Balint principles, for psychiatry registrars working in forensic settings across Victoria.

**Method:**

We describe the process of setting up a Balint group for this professional population. Qualitative feedback was obtained from group members. The data was analysed thematically, regarding motivating factors for group attendance and feedback post pilot group.

**Results:**

Overall, feedback on the pilot Balint group was positive, which led to the group being extended for recurring 6-month periods. We share an illustrative example of a complex case which could be formulated in a forensic psychiatry Balint group setting, alongside reflections of the facilitators of the group.

**Conclusions:**

Reflection is paramount for safe, effective mental health treatment, particularly in the context of forensic psychiatry. Our pilot results highlight areas where the approach of setting up a fledgling Balint group has been successful. We hope to inspire others to engage and participate in routine reflective practice.

## Introduction

It is recognised that treating mentally unwell people can be emotionally challenging, contributing to stress and burnout in medical workforces.^1^ Elements of such relationships may not be obvious unless clinicians stop to think.^2^ Such dynamics may be amplified when clinicians work intensively with long-term patients such as in forensic secure services.^3^ Those working in such settings can encounter ethical challenges that are less common in general mental health settings, as well as more frequent incidents of verbal aggression and physical violence.^4^ In forensic mental health services, it is paramount that clinicians remain aware of the judiciary as an omnipresent influence, representing punishment as well as protection of society. In relational terms, this could be viewed as a third ‘person’ in the patient-doctor relationship, with the potential of introducing coercive or punitive element to patient care. It could be suggested that a patient who discharges violent impulses against staff may in part be displacing the anger they feel against these elements. This is one example of the many psychodynamic principles that staff members working in this setting may reflect on.^5^

Reflective practice groups are a commonly used tool for facilitating staff reflection on their interactions with patients and each other. Such groups have several functions for staff: support to reduce stress and burnout; to understand and process clinicians’ responses to patients; to reflect on possible unhelpful enactments of transference reactions; and to support the team to work well together and sustain consistent, caring responses to patients.^6^ One model of reflective practice is that of a Balint group^7^ which is used primarily by psychiatrists and general practitioners, is grounded in psychoanalysis, and has the intention of bringing to awareness unconscious processes in the doctor–patient relationship such as transference, countertransference and unconsciously motivated acts. Balint groups are regular meetings – usually weekly or fortnightly – where a small group of professionals can come together.

The groups are led by one or two facilitators, who aim to enable dialogue between group members and encourage reflection, without imposing an agenda. Group members are encouraged to discuss challenging and/or emotive situations they have encountered at work, surrounding an interaction with a patient, and the overlay of other carers/systems involved with the patient, especially from a relational perspective. The group should be a confidential, non-judgemental scenario, where staff are not afraid to express any views they may have.^7^ It is important that such a group is voluntary, as in this type of work group members should come to the group willing to share their experience and not under duress.^7^

Within our organisation, there was a structured reflective practice programme set up for all members of the multidisciplinary team, with positive results reported during a recent analysis of this.^4^ Such multidisciplinary groups certainly have their place; however, it was our belief that, alongside such groups, it is also of benefit to have profession specific groups where cases can be considered from a more focused perspective.

We describe our experience of setting up a Balint group for psychiatry registrars within Forensicare (The Victorian Institute of Forensic Mental Health), who are responsible for providing adult forensic mental health services in Victoria. This would be the first reflective practice group to be specifically targeted at forensic psychiatry registrars within the state. We describe the process of setting up this group. We detail qualitative results of two surveys which focused on participants’ motivation for joining the pilot Balint group, as well as feedback on the group, undertaken at the start and end points. We describe further development of the group, challenges encountered, and an example of how a case may be formulated in a forensic psychiatry Balint group setting.

## Methods

In January 2021, the authors formulated a proposal to send to the executive director of clinical services within Forensicare. After a period of discussion considering service needs, staff members within the organisation and their own learning and supervision, two authors (GW and RB) proposed a plan to commence Balint Society of Australia & New Zealand (BSANZ) leadership supervision.^8^ JS already had a wealth of experience in reflective practice group leadership and subsequently provided leadership alongside GW. Group implementation plans were discussed at Balint leadership group supervision, allowing discussion with several other Balint group leaders, who provided their own expertise.

An email for expressions of interest was sent out to a total of 31 registrars within the organisation, who were working across several different settings including inpatient, multiple prison locations spread across Victoria and community posts within Melbourne. The decision was made to run the initial pilot group via Microsoft teams, with an aim of increasing access to the group between registrars working independently from each other, but also because of ongoing Coronavirus disease restrictions at that time.

We conducted a qualitative survey of registrars who agreed to sign up, prior to commencing this pilot Balint group. This was distributed via email, and responses were received between 18th August and 28th September 2022. The survey focused on the question of participants’ motivation for signing up for the group, as well as whether they had prior experience of attending a Balint group. A subsequent qualitative survey was carried out around the ending point of the pilot group, to gain feedback on group members’ experiences, with responses received between 24th November and 2nd February 2022.

The introductory paragraph to the survey contained an explanation for its rationale, followed by a clear statement verifying that the identity of clinicians submitting responses to the survey would remain anonymous, and that collated results would be distributed via appropriate forums including presentation and/or publication. Participants had the option to decline to be surveyed if they disagreed with this, and by commencing the survey, informed consent was confirmed by participants. Open-ended responses were analysed using thematic analysis, where data was coded and subsequently grouped into various themes, with authors deriving a common consensus.^9^

## Results

11/31 (35.5%) of registrars within Forensicare voluntarily took part in the pilot registrar Balint group. The pre-group survey was filled in by all 11 group members. Most had no prior experience of Balint groups (*n* = 6, 54.5%), and prior to initiation of the group, no group member had the opportunity to be part of a Balint group within working hours at Forensicare.

### Motivation for attending the Balint group

Four themes were identified regarding motivation for attending the Balint group (see Table 1): interest in developing psychotherapy skills (*n* = 6), desire for peer review and supervision (*n* = 4), improved support with more challenging clients (*n* = 4) and previous positive experience of reflective groups (*n* = 2).

**Table 1.** Motivating factors for joining Balint Group

Theme	Clinician quotes	Theme mentioned by (*n*)	%
Interest in developing psychotherapy skills	‘Gain first-hand experience in Balint group’ ‘I have really enjoyed psychodynamic clinical discussions and reflecting on how principles such as transference and countertransference impact on patient care. I think this is particularly relevant in forensic practice when clients may have done things that generate negative emotions’. ‘To enhance thinking about patients and systems in a psychodynamic/psychotherapeutic manner’ (*n* = 4)	6	54.5
Desire for peer review and supervision	‘I like the experience of sharing peers’ views in managing complex cases’. ‘Keen to be involved in more peer-related activities and group discussions’ ‘Professional development, becoming a more thoughtful clinician and to further understand others’ perspectives’. (*n* = 2)	4	36.4
Improved support with more challenging clients	‘Access to support for difficult patients’ (*n* = 2) ‘Keen to be involved in group discussions regarding patients/difficult scenarios arising at work’. ‘They offer an opportunity to reflect on challenging clinical encounters/relationships in a safe space. Learn to better understand my own emotional responses and how that fits with clinical work’.	4	36.4
Previous positive experience of reflective groups	‘Engaged in one during psychotherapy lectures. Surprised at how useful I found it and have wanted to join one’. ‘I was a member of an informal psychodynamic discussion at (previous health service). We discussed patients but also teams and systems, I think it helped me be more reflective’.	2	18.2

Table 1 shows qualitative responses to questioning on motivating factors to join the Balint group, with answers grouped into varying themes. The number of respondents relating to each theme is detailed, alongside the percentage of total respondents.

### Feedback from group members around the end point of the pilot

Six end point surveys were received. Three main themes were identified in regard to qualitative feedback: barriers to attendance (*n* = 3), benefits of the group (*n* = 6) and suggestions for improvement (*n* = 4). Some group members mentioned that they were struggling to attend some groups despite wishing to, due to other commitments such as clinical workload. There was positive feedback that group attendance helped clinicians make sense of complex patient interactions, and improved team working (see Table 2).

**Table 2.** Feedback on Balint group

Theme	Clinician quotes	Theme mentioned by (*n*)	%
Barriers to attendance	‘Clinical reviews (at clashing time) and shared office with audio/visual limitations’. ‘Having double-booked appointments at the same time; but sometimes cannot be avoided as things cannot be shifted’. 'Work related commitments’	3	50
Benefits of group	‘Allowed to reflect and think about approaches in my day-to-day clinical work. Also allows a space to experience vicariously, other clinical colleagues experiences in their clinical work’. ‘Good to hear other practitioner’s scenarios and listen to all the comments, which has been helpful in my clinical work’. ‘Helpful to hear from others who have varied experience in psychiatry and the forensic system. Importantly, to feel validated and not so alone with difficult feelings e.g. hopelessness, therapeutic’ ‘Highly valuable space to be able to reflect with colleagues from a similar background and training but from a different part of the service. It has provided heterogenous perspectives that will be useful for future self-reflection’. ‘Improved thinking around situations where… unable to diffuse the situation, and have to end up terminating the interview session’ ‘Thank you for facilitating such a group. I wonder why it isn’t done more widely?!’	6	100
Suggestions for improvement	‘If we can have a roster to present cases and if we can have a chance to go through a summary of cases in advance to the meeting we can get ready for the discussion well’ ‘I really value the consistency of the group members to be able to safely share ideas, therefore smaller group may be helpful’. ‘(Help with trying to).. find a private office and to let the consultant know ahead of time’. ‘Would definitely prefer being in a room together but coronavirus (pandemic restrictions) are here to stay unfortunately’	4	66.7

Table 2 shows qualitative responses to questioning on feedback on the pilot group, with answers grouped into varying themes. The number of respondents relating to each theme is detailed, alongside the percentage of total respondents.

### Authors’ reflections on challenges encountered

There were some challenges in implementation, with learning points to reflect upon. Setting up a group such as this, which involves multiple organisations and professionals, requires a careful balancing act, in that each organisation and individual may have different expectations and goals. Setting up a Balint group and securing funding and support for this requires careful negotiation between parties, and ultimately may not be possible in some less supportive or understanding environments. It can be challenging to find a suitable time for running a group, as this requires careful balancing of organisational requirements, alongside leaders and group members already scheduled commitments. There is a level of compromise and subsequent considered discussion required from all parties.

The two main issues that were encountered when running groups were issues related to audio-visual connection on Microsoft teams – due to online security restrictions this is unfortunately a common issue particularly for those working in a prison environment. The other more predominant issue was the Balint group clashing with other commitments such as unit clinical review or annual leave. The Coronavirus also had an impact on group members either being on sick leave whilst isolating or having to cover for other colleagues doing the same. Due to absences, several groups had to be cancelled at short notice due to lack of attendees.

### Continuation of the group post the successful pilot

Following the pilot Balint group which ran from October 2021 to January 2022, further liaison was had with the executive director of clinical services and group members. Due to the reported success of the group, it was agreed that funding would continue to be provided for GW to attend Balint leadership training. The group was therefore extended on from February to August 2022 (with 10/29 registrars as group members), and subsequently extended for another 6-month block starting August 2022 (with 9/33 registrars as group members). An example of how a case could be formulated in a Balint group carried out within a forensic psychiatry setting is described in the following case presentation.

### Case presentation

It should be noted that this is a fictional case, used to demonstrate examples of topics which were presented and discussed in our Balint group.

Ms W is a 50-year-old female patient, cared for on an inpatient forensic psychiatry rehabilitation unit. She is morbidly obese (with a body mass index of 60), and has poorly controlled diabetes, as well as a diagnosis of psychotic illness. She has a history of witnessing domestic violence, as well as being the victim of sexual assault from an ex-partner. Childhood attachment difficulties are documented, with a lack of expressed emotional care and physical abuse.

Ms W engaged in a pattern of offending, including distribution and use of illicit substances, and serious assault on a neighbour. She was incarcerated and subsequently transferred to an inpatient forensic psychiatric facility due to concerns around her mental health. On the rehabilitation unit, there were repeated episodes of Ms W providing misinformation to staff, for example, by secretly eating unhealthy snacks whilst in the kitchen. She would deny she has diabetes and intermittently refuse prescribed medical treatment for this. She would often be reviewed in bed as she refused to attend the interview room, and on attempts would commonly roll over and disengage.

The registrar who presented this case suggested that they feel that Ms W does not want to leave hospital and there is a communal feeling of lack of therapeutic optimism, posing the question to other group members of how they might feel about such a presentation.

### Follow-up questions posed by group members

• How much of the patient’s presentation is within her control?

The impact of childhood trauma on resultant development of personality factors was considered, including impulsivity and poor emotional regulation. Consumption of food (and illicit substances prior to admission), could represent a form of self-soothing and self-regulation. The potential of experience of childhood abuse being a contributing factor in making Ms W overeating to make herself ‘unattractive’, as a way of defending herself against male attention, was also raised. The potential impact of antipsychotic medication on weight gain was also noted.

• What is driving the patient’s behaviour?

Factors such as shame or boredom driving a cycle of eating and weight gain were raised, as was the hypothesis that Ms W could be medicalising her eating difficulties in order to alleviate a sense of self blame. Another suggestion was whether Ms W could be making herself ill in an effort to get a break from the psychiatric unit and be transferred to a medical hospital, engaging in a form of abnormal illness behaviour. Finally, it was suggested that if she were physically unwell, this could lead to physical contact from the treating team, by way of being physically handled for examination, monitoring, or moving/transferring to a general hospital. Within forensic secure units, physical contact between staff and patients is generally prohibited, and Ms W’s behaviours could be representative of attachment/contact seeking behaviour.

• What aspect of this case is causing frustration in staff?

The group commented that staff’s lack of understanding of potential unconscious and psychologically motivated behaviours could be driving Ms W’s presentation. This led to counter transferential reactions of frustration and annoyance at her non-engagement in treatment. It was also felt that perhaps Ms W’s helplessness within the circumstances she found herself in was being unconsciously projected onto the treating team who identified with this helplessness and felt out of control. A more conscious and defensive reaction by staff could have reflected the need for safety for themselves such that the patient did not suddenly and significantly deteriorate from a medical perspective on the unit, with potential for an internal investigation for inadequate and negligent care.

Overall, in discussing this case there was a feeling that the Balint group members had become more aware of the unconscious and psychological meaningfulness of Ms W’s behaviours, making them more sympathetic towards her and the predicament she found herself in.

### Follow-up work

Following our group being set up, the Royal Commission into Victoria’s Mental Health System^10^ recommended that all junior doctors in Victoria have at least one rotation in psychiatry, where they have access to fortnightly reflective practice groups (with specific reference to Balint Groups). This provided positive acknowledgement that we had helped the organisation meet this requirement before it was announced.

The authors continued to seek feedback from group members regarding potential improvements for the group, and at the time when all authors had stopped full time work at Forensicare in February 2023, the intention was for the group to continue long-term. More recent improvements made upon commencing the August 2022 group (following comments from group members) included running the groups at exactly two-week intervals to ensure consistency and ensuring a firmer stance on group members attending regularly to improve the feelings of group safety. When considering the pilot group feedback detailed in Table 2, the suggestion of having a roster of case presentations was discussed but not implemented, as it was felt this would be against the ethos of Balint Group presentation, where cases should be presented briefly, informally and without notes. This helps bring the presenters’ feelings, reactions and associations to the forefront, rather than specific clinical details.^7^ Leadership transition plans were made to maximise the chances of the group continuing to run successfully. This included drawing up a plan of potential future group leaders within the organisation and providing advice and recommendations to the executive director of clinical services.

## Discussion

We believe that our results add to the limited but growing evidence of the benefits of reflective practice groups in the forensic psychiatry setting.^4,11^ Previous research has suggested that benefits of Balint groups for mental health professionals working in the forensic psychiatry setting specifically include increased team cohesion and appreciation for the psychodynamic formulation of a client.^11^ Previous research has identified the factor of competing clinical demands impacting group attendance.^4,11,12^ These results have correlation with our findings. The importance of ‘buy in’ from senior management as well as appropriate coverage for staff to attend reflective practice groups has also been highlighted in previous research, which ties in with our reflections.^4^

We acknowledge the low response rate to our survey questionnaire, however, still believe that this gives a provisional snapshot of feedback from participants in our Balint group. As with all survey-based research, there is a potential risk of bias in that a certain subgroup of clinicians who have strong feelings about the group are more likely to respond. Therefore, our results may overestimate overall levels of participant satisfaction. Future research should aim for improved response rates, considering methodologies such as modest rewards for participation, for example. It may be beneficial to assess participants’ reflective abilities within such a group using a more structured and validated questionnaire.^13^ Another limitation of this piece of work is that the authors have evaluated their own work – a potential source of bias.

Based on our qualitative results of group feedback and our own reflections as group leaders, we identified multiple areas of benefit of this Balint group. Staff who participated in such groups appeared to increase their skill levels in dealing with complex clients from an emotive perspective. This could improve the relationship with clients, increase compassion and deepens understanding via peer reflection. Clients may have benefitted from staff being able to understand their own thoughts, needs and behaviours better. The Balint group may have allowed greater understanding of the client to be seen more clearly as the individual human being that they are. Organisational benefits observed included more widespread growth of staff skills via a natural structure for mentoring and peer feedback. This hypothetically could reduce staff stress, burnout and corresponding absence. The requirement for an appropriate staff wellbeing strategy is now enshrined in occupational health and safety standards,^14^ so the implementation of such a group could help in satisfying this.

It is part of the (United Kingdom’s) Royal College of Psychiatrists’ core learning curriculum for psychiatry trainees that trainees will attend regular reflective practice groups during their training.^15^ Usually, this requirement is achieved by weekly attendance at reflective practice groups that typically model Balint principles for doctor-patient discussions. Before a UK psychiatry trainee is permitted to start providing supervised psychological therapy (also a requirement of training), the trainee must have attended a regular reflective practice group for a period of at least a year.^15^ Whilst opportunities for joining reflective practice groups do appear to vary between organisations, it anecdotally appeared that psychiatry trainees in Australia have less opportunities in this field, without a stated curriculum requirement for engaging in a specific number of sessions.^16^ The authors suggest that it may be of benefit for Australian psychiatry trainees if attending a reflective practice and/or Balint Group was a routine requirement for psychiatry registrar training.

For professionals in other services considering setting up their own Balint group, we would recommend liaison with BSANZ, for advice around engagement in leadership training and to help ensure that the Balint model is followed within new groups. Without such input, there is a risk that a group may be referred to as a Balint group but use non-methodologically tested techniques which could limit a positive experience for participants. Skill sets of potential group leaders should be considered. For example, in our group we found it beneficial to have the group led by one consultant with extensive experience in psychotherapeutic work (JS), and a psychiatric registrar who was engaged in BSANZ leadership training (GW). This seemed a complementary approach. Peer feedback between leaders is also useful in receiving and sharing advice on positive and negative experiences in group development. Finally, we found it beneficial to involve influential figures from our organisation to promote the group, for example, the local clinical director and lead registrar.

## Conclusion

We successfully established the first reflective practice group for forensic psychiatry registrars in the state of Victoria, running this for an 18-month period. We received positive feedback for this, and as a result planned for the group to continue into the future. Reflection is essential for safe, effective psychiatric care, particularly in the context of forensic psychiatry. Our pilot results give an example of some areas where the approach of setting up a fledgling Balint group has been effective. It appears that this was beneficial for group members in helping them develop their reflection and empathy capabilities, which may ultimately improve patient care. We hope to inspire others to engage and participate in routine reflective practice.
